# Investigation of Melatonin Incorporated CMC-Gelatin Based Edible Coating on the Alleviation of Chilling Injury Induced Pericarp Browning in Longkong

**DOI:** 10.3390/foods13010072

**Published:** 2023-12-24

**Authors:** Karthikeyan Venkatachalam, Narin Charoenphun, Somwang Lekjing, Paramee Noonim

**Affiliations:** 1 Faculty of Innovative Agriculture and Fishery Establishment Project, Prince of Songkla University, Surat Thani Campus, Makham Tia, Mueang, Surat Thani 84000, Thailand; karthikeyan.v@psu.ac.th (K.V.); somwang.s@psu.ac.th (S.L.); 2 Faculty of Science and Arts, Burapha University Chanthaburi Campus, Chanthaburi 22170, Thailand; narinch@buu.ac.th

**Keywords:** longkong pericarp, chilling injury, melatonin, CMC-gelatin, storage, enzyme activity, fruit quality

## Abstract

Longkong (*Aglaia dookkoo* Griff.) fruit is prone to rapid pericarp browning and shortened shelf life (<7 days) under prolonged low-temperature storage. This study investigates the effect of an edible coating, comprising carboxymethyl cellulose (CMC) and gelatin in a fixed 3:1 ratio, integrated with various concentrations of melatonin (MT) (0.4, 0.8, and 1.2 mM/L) to mitigate chilling injury in longkong fruit. Coated longkong fruits were stored at 13 °C with 90% relative humidity for 18 days and underwent physicochemical evaluations every three days. Samples coated with CMC-Gel without MT and uncoated fruits were served as controls. The findings indicated that the CMC-Gel-MT coating significantly mitigated pericarp browning, chilling injury, weight loss, and respiration rate increase under extended cold storage conditions. High concentrations of MT (≥0.8 mM/L) in the coating notably inhibited the activities of cellular degrading enzymes such as lipoxygenase and phospholipase D. This inhibition contributed to reduced membrane permeability, lower reactive oxygen species accumulation (H_2_O_2_, OH^−^, O_2_^−^), and decreased malondialdehyde levels in the longkong pericarp. Furthermore, the CMC-Gel-MT coating increased the activity of phenylalanine ammonia lyase, leading to an enhancement in phenolic content. Consequently, it improved the fruit’s ability to scavenge DPPH (2,2-diphenyl-1-picrylhydrazyl) and ABTS (2,20-azino-di-3-ethylbenzthiazoline sulfonic acid) radicals. Control samples exhibited high levels of pericarp browning-related enzymes (polyphenol oxidase, peroxidase), whereas CMC-Gel-MT-coated fruits, particularly at higher MT concentrations, showed significant reductions in those enzyme activities. In conclusion, incorporating high concentrations of MT in a CMC-Gel-based edible coating is a promising alternative for mitigating chilling injury in longkong fruit.

## 1. Introduction

Longkong (*Aglaia dookkoo* Griff.) is a non-climacteric tropical fruit, abundant in health-beneficial phytonutrients, that demonstrates potent antioxidant and antimicrobial properties [1]. Additionally, the fruit is characterized by a unique flavor profile, primarily recognized for its pleasant taste, which starts sweet and then shifts to a mild sourness [2]. Although the fruit offers significant health benefits, only the flesh is edible, while other parts, including the seed and pericarp, are nonedible. The fruit typically contains 3–5 green seeds enveloped in white, pale flesh. It is recommended to be cautious while consuming longkong fruit, as inadvertent ingestion of the seeds may lead to an adverse sensory experience attributable to their pronounced bitterness [3]. The pericarp, in contrast, is tasteless. However, both pericarp and seeds have been utilized in various traditional medicines, and recent studies suggest they may contain key components useful in modern drug production [4]. Longkong fruit is rich in essential nutrients, but its short shelf life limits its market reach, consequently diminishing its economic value [5]. Predominantly grown in Thailand and other tropical countries in Southeast Asia, the fruit suffers from rapid deterioration due to browning and microbial spoilage, which has led to limited recognition and restricted market coverage. Several studies have been conducted to investigate the causes of spoilage, with rapid pericarp deterioration, mainly browning and consequently a short shelf life, typically under ambient storage conditions (less than 5–7 days) or chilling temperatures (less than 5 days), being the primary issues [6,7,8]. To expand its market reach, longkong fruit must be chilled for transportation over long distances, yet this low-temperature storage can exacerbate chilling injuries [9]. Additionally, environmental variables may contribute to the extrinsic aging of longkong fruits. This causes the production of reactive oxygen species (ROS), which is thought to be an essential indicator of longkong pericarp resiliency to solar ultraviolet radiation and chilling injury. In terms of chilling injury, the ROS are produced and accumulate when cold stress occurs to longkong fruits after harvest. This initiates an oxidative chain reaction that leads to oxidative damage and loss of function in the cell membrane [4,6]. Numerous postharvest studies have explored ways to extend longkong fruit’s shelf life, employing various active and passive modified atmospheric packaging techniques as well as phytohormone treatments to control chilling injury [6,10,11]. While some of these methods are easy to use, they may struggle to maintain quality, and vice versa. This situation presents an ongoing opportunity for research into longkong fruit, aiming to find a feasible method to achieve extended shelf life with technology that is both easy to use and effective for commercial purposes.

Edible coatings, specifically those involving polymers, are a prevalent postharvest technique used to extend the shelf life of a broad array of plant produce, especially fruits [12]. Carboxymethyl cellulase (CMC), a polysaccharide-based edible polymer, boasts commendable coating properties due to its pronounced hydrophilicity. Notably, CMC can be integrated with various functional ingredients, which enhances its efficiency in preserving fruit quality and extending shelf life. Additionally, this polymer can form a colorless, odorless, nontoxic, stable, and uniform coating, although it sometimes lacks sufficient mechanical strength [13]. To address this, recent studies have highlighted the potential of blending polymers to bolster the coating’s strength and prevent fruit quality loss [14]. Gelatin (Gel), a protein-based edible polymer, has gained popularity due to its biodegradable attributes and superior film and coating formation capabilities [15]. Combining CMC and Gel has demonstrated significant potential in prolonging the shelf life of agricultural products, with an optimal concentration blend reported at a 3:1 ratio [16]. The combined use of CMC and Gel offers an ecofriendly, biodegradable alternative for edible food coatings [17,18]. This blend exhibits increased viscoelasticity, resistance to oil/fat, enhanced coating properties, and a proven capability to prolong fruit shelf life [17]. Furthermore, the CMC–gelatin biopolymeric matrix can produce transparent edible coatings and serve as a carrier for active ingredients, including phytohormones, antioxidants, and antimicrobial agents [19]. Recent studies have shown that the addition of phytohormones such as melatonin (MT) into the coating emulsion can offer immense advantages, including enhanced disease resistance, antioxidant activity, resistance to pathogens, decay inhibition, and extended fruit shelf life [20]. MT is renowned for its protective effects against cellular and DNA damage, and its role in inhibiting stress-induced peroxidation enhances oxidation resistance in plants [21]. It mitigates pericarp browning, onset of free radicals, reactive oxygen species, and phenolic oxidations. Moreover, MT has been shown to control damage from extended chilling and suppress lipoxygenase activity, which in turn manages alterations in cell membrane fatty acids [22]. The efficacy of MT in postharvest fruit treatments varies with the concentration used and its effectiveness also varies among fruit types [23]. When MT was applied exogenously, it dramatically improved disease resistance and decreased decay in bananas, grapes, kiwis, strawberries, and plums [20].

The present study utilized the opportunity to use of this unique combination including CMC-Gel coating that incorporated various concentrations of MT to investigate the effects of controlling pericarp browning in longkong fruit stored under prolonged chilling temperatures by regulating ROS and the membrane metabolism.

## 2. Materials and Methods

### 2.1. Raw Materials, Chemicals, and Reagents

Longkong fruit samples were collected from a designated garden in the Nasan District, Surat Thani Province, Thailand. The fruit samples were gathered 14 weeks post-anthesis, after which they were assessed based on the maturity index (color, total soluble solids, and titratable acidity) recommended by Venkatachalam and Meenune [7]. The collected fruits were transported to the laboratory within 4–6 h for further inspection to identify any visible damage caused by abrasion, impact, or microbial attack. Once the fruits were selected for treatment, they were individually detached from their bunches with the raceme end still attached and washed with distilled water to remove any dust or dirt on the surface and submerged in sodium hypochlorite solution (200 µL/L) for 2 min, followed by washing again with distilled water. Subsequently, excess moisture on the fruits was eliminated with paper towels, followed by exposure to an electric fan to ensure the complete removal of any residual moisture on their surface. The fruits were then prepared for the coating application, as detailed in Section 2.3.

For coating, food-grade chemicals and reagents such as CMC, gelatin, melatonin, and glycerol were used, while all other chemicals utilized for analyses in this study were of analytical grade. All the chemicals and reagents used for this study were procured from Sigma Aldrich, Bangkok, Thailand.

### 2.2. Formulation of Coating and Treatment

**Figure 1:**
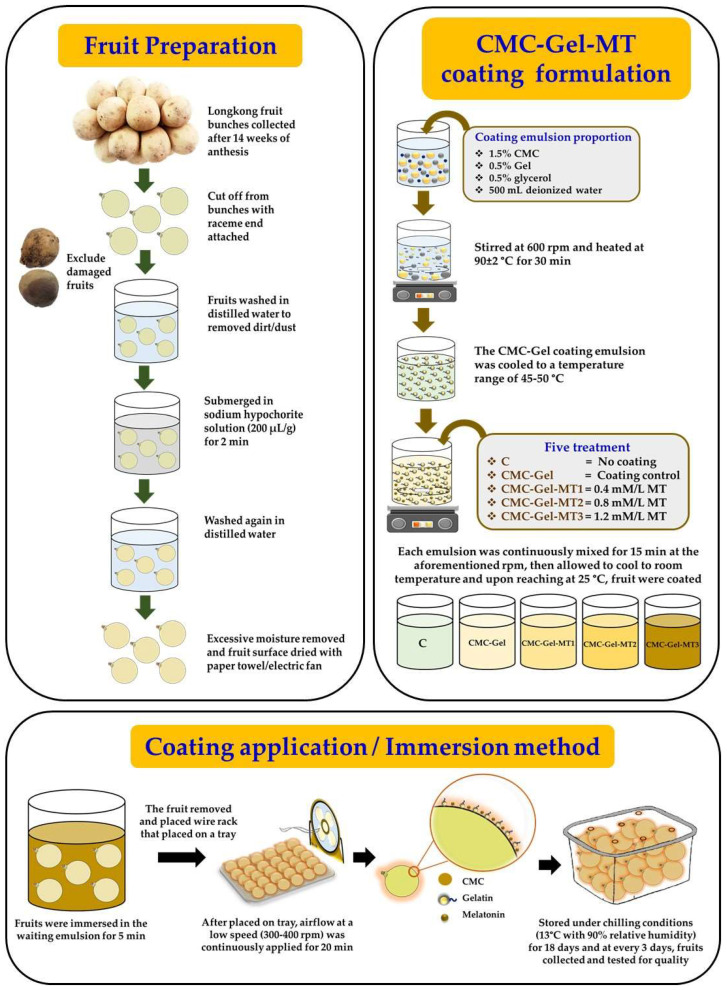
Infographic presentation of raw material preparation, coating formulation and application on longkong fruit surface.

The CMC-Gel coating emulsion was prepared following the method described by Vargas-Torrico et al. [24], with certain modifications. The emulsion, consisting of 1.5% CMC, 0.5% Gel, and 0.5% glycerol, was mixed in 500 mL of deionized water. This mixture was continuously stirred at 600 rpm and heated on a magnetic stirrer hot plate at 90 ± 2 °C for 30 min. After this, the emulsion was cooled to a temperature range of 45–50 °C. MT was then introduced separately in four different concentrations: 0.4, 0.8, and 1.2 mM/L, resulting in treatments CMC-Gel-MT1, MT2, MT3, respectively. The CMC-Gel coating emulsion without MT was served as treatment–control and fruit with no coating was served as control (C). Each emulsion was continuously mixed for 15 min at the aforementioned rpm, then allowed to cool to room temperature. Upon reaching approximately 25 °C, the emulsions were applied to the fruits using an immersion method. The prepared longkong fruits were divided into five groups, with 30 fruits each, for every replication per treatment group. They were meticulously immersed in their respective emulsions, ensuring a uniform coating. The fruits were then subjected to airflow at a low speed (approximately 300–400 rpm) for 20 min to dry the surface. Once completely dried, the fruits were placed in perforated punnets and stored under chilling conditions (13 °C with 90% relative humidity) for 18 days. Every three days, the fruits were assessed for various quality attributes, as outlined in Section 2.4. Figure 1 shows the infographic representation of raw material preparation, CMC-Gel-MT coating formation and application on longkong fruit.

### 2.3. Quality Analysis

#### 2.3.1. Pericarp Color and Observation of Fruit Surface Appearance

To quantify the fruit’s color properties, this study measured 15 individual fruits from each replicate. A colorimeter (HunterLab, Reston, VA, USA) was employed to determine the lightness (L*), redness (a*), and yellowness (b*) color parameters of the fruit’s pericarp (outer skin) by following the procedure proposed by Charoenphun et al. [6]; four random surfaces on each fruit were selected for analysis. For the observation of fruit surface appearance, the fruits from initial and final day of storage were carefully collected—any surface coating and moisture removed—and digitally photographed using a handheld Sony digital camera (Model ZV-1F, Tokyo, Japan), following the method of Venkatachalam [25].

#### 2.3.2. Chilling Injury Index (CI)

To assess CI in longkong fruit pericarp (outer skin), this study employed visual inspection by following the method of Nguyen et al. [26]. Longkong fruit develops a distinctive brown discoloration on its pericarp when chilled, which we quantified using a 5-point scale ranging from 1 (no CI) to 5 (severe injury).

#### 2.3.3. Electrolytic Leakage (EL)

EL measurement in longkong fruit pericarp was adapted from the method of Wang et al. [27]. Twenty longkong pericarp discs from each replication were cut out using a metal borer. The discs were then rinsed with distilled water and air-dried on absorbent paper. Each disc was placed in an individual test tube holding 20 mL of distilled water. These tubes were then shaken in a water bath at 25 °C for half an hour. Then, they were measured initial conductivity (L0) with a conductivity meter and then the tubes were subjected to a temperature of 100 °C for 20 min, followed by rapid cooling. Post-cooling, the pericarp discs were measured for final electrical conductivity (L1). The rate of electrolyte leakage in longkong fruit was calculated as the percentage ratio of L0 to L1 ((L0/L1) × 100%).

#### 2.3.4. Malondialdehyde (MDA) Content

MDA content in longkong fruit pericarp was assessed using the procedure proposed by Noonim and Venkatachalam [28]. Two grams of pericarp was blended with 8 mL of sodium phosphate buffer (50 mmol/L, pH 7.8) and then the mixture was centrifuged at 12,000× *g* and 4 °C for 15 min. Two milliliters of the resulting supernatant was collected and combined with 3 mL of thiobarbituric acid solution (5 g/L) in 0.1% trichloroacetic acid (TCA). Then, this mixture was heated in a boiling water bath for 15 min, quickly cooled, and centrifuged under the same conditions as above. The absorbance of the final supernatant was recorded at wavelengths of 450, 532, and 600 nm. The MDA levels were determined, and the findings of the samples were reported as nmol/g of fresh weight (FW).

#### 2.3.5. Fruit Weight Loss

The weight loss of longkong fruit was determined by following the method of Chen et al. [29]. This involved daily weighing of the fruit samples and calculating the weight loss over time by comparing the weights at different storage intervals. The data were then presented as percentage of weight loss.

#### 2.3.6. Respiration Rate

The respiration rate of longkong fruit was assessed using the method of Caleb et al. [30]. For analysis, 30 fruits for each replication were collected and placed in individual 3 L sealed glass chambers. These chambers were then kept at room temperature for 3 h. Subsequently, a 1 mL gas sample was extracted with a syringe and analyzed using a gas chromatograph (GC; Auto systems XL, Perkin Elmer, Waltham, MA, USA), which was equipped with a Porapack Q column (80/100 mesh) and a thermal conductivity detector. The respiration rates were quantified as mL CO_2_ per mg kg^−1^ h^−1^.

#### 2.3.7. Determination of Hydrogen Peroxide (H_2_O_2_)

H_2_O_2_ content in longkong fruit pericarp was assessed by using the method of Patterson et al. [31]. A 2 g pericarp sample was homogenized in 15 mL of acetone under cold conditions and then the mixture was centrifuged at 6000× *g* for 15 min at 4 °C. After that, 1 mL of the supernatant was added along with 0.1 mL of 5% TiOSO_4_ and 0.2 mL of NH_3_ to the centrifuge tube and then centrifuged again under similar conditions for 10 min. The resultant pellet was then dissolved in 3 mL of 10% H_2_SO_4_ and centrifuged at 5000× *g* for 10 min at 4 °C. Then, the final supernatant was used to measure the H_2_O_2_ content. The measurement was based on the absorbance at 410 nm, using a UV-Vis spectrophotometer (model: Mini UV 1240, Shimadzu, Kyoto, Japan). The results were then expressed as nmol g^−1^ FW.

#### 2.3.8. Determination of Hydroxyl Radical (OH^−^)

The OH^−^ content in longkong fruit pericarp was assessed using the 2-deoxyribose oxidation method by Chung et al. [32]. A 10 g pericarp sample was homogenized in 50 mL of methanol, followed by filtration and centrifugation at 2000× *g* for 10 min at 4 °C. The supernatant obtained was then used for analysis. In a test tube, 0.2 mL of a reagent mixture containing 10 mmol of iron (II) sulfate heptahydrate and 10 mmol of ethylenediaminetetraacetic acid (EDTA) was combined with 0.2 mL of 2-deoxyribose. After thorough mixing, 0.8 mL of the sample was added to this mixture, along with 1 mL of 0.1 mol sodium phosphate buffer at pH 7.4, followed by the addition of 200 µL of 10 mmol hydrogen peroxide to the mixture, which was then incubated at 37 °C for 4 h. Subsequently, 1 mL of 2.8% trichloroacetic acid (TCA) solution was added, and the mixture was heated in a boiling water bath for 10 min before cooling to room temperature. The absorbance of the mixture was measured at 520 nm using a UV-Vis spectrophotometer (model: Mini UV 1240, Shimadzu, Kyoto, Japan). The results were then expressed as nmol g^−1^ FW.

#### 2.3.9. Determination of Superoxide Anion (O_2_^−^) Radical

To measure the O_2_^−^ content [33] in longkong fruit pericarp, a 4 g pericarp sample was homogenized under cold conditions (4–8 °C) in 12 mL of 50 mmol/L potassium phosphate buffer (pH 7.8) with 1% polyvinylpyrrolidone. The homogenate was then centrifuged at 5000× *g* for 15 min at 4 °C. Subsequently, 1 mL of the resulting supernatant was combined with 0.9 mL of the same potassium phosphate buffer and 0.1 mL of 10 mmol/L hydroxylamine hydrochloride and this mixture was thoroughly mixed and incubated at 25 °C for 30 min. Following this, 1 mL of the incubated mixture was added to 1 mL of 17 mmol/L metanilic acid and 1 mL of 7 mmol/L 1-naphthylamine. After thorough mixing, this final mixture was incubated again at 25 °C for 30 min. The absorbance of the mixture was then measured at 530 nm using a UV-Vis spectrophotometer (model Mini UV 1240, Shimadzu, Kyoto, Japan). The results were then expressed as nmol g^−1^ FW.

#### 2.3.10. Total Phenolic Content (TPC)

The determination of TPC in longkong fruit pericarp was performed using a colorimetric method [34]. Five grams of the pericarp was homogenized with 25 mL of 95% ethanol, the mixture was centrifuged at 12,000× *g* and 4 °C for 15 min, and the supernatant was collected and proceeded for the TPC measurement. The procedure involved mixing 8.4 mL of distilled water with 100 μL of the fruit sample and 500 μL of Folin–Cioclateu reagent in a test tube. This mixture was left to stand for 3 min, after which 1.0 mL of 20% sodium carbonate was added. The mixture was then thoroughly vortexed (Vision Scientific Co., Ltd., Daegu, Republic of Korea). After an hour, the absorbance at 720 nm was measured using a spectrophotometer (model: Mini UV 1240, Shimadzu, Kyoto, Japan). The phenolic content was quantified by comparing the absorbance to a standard curve created with chlorogenic acid, and the results were expressed as µg gallic equivalent (GAE) g^−1^ FW.

#### 2.3.11. Antioxidant Activities

For determining the antioxidant activities, a 5 g sample of pericarp was homogenized with 25 mL of 95% ethanol, the mixture was centrifuged at 12,000× *g* and 4 °C for 15 min, and the supernatant was collected and processed for antioxidant measurement. For DPPH radical scavenging activity [35] of longkong fruit, a 100 µL sample of the extract was thoroughly mixed with 3.9 mL of 60 µmol/L DPPH solution in a test tube. This mixture was then left to incubate for 30 min in darkness at room temperature. The absorbance was measured at 515 nm using a spectrophotometer (model: Mini UV 1240, Shimadzu, Kyoto, Japan). The results were expressed as percentages. For ABTS radical scavenging activity [36], a 100 µL sample of extract was mixed with 100 µL of ABTS reagent, and then this mixture was incubated for 6 min at ambient temperature. After that, the absorbance readings were taken at 734 nm using a spectrophotometer (model: Mini UV 1240, Shimadzu, Kyoto, Japan). The results were expressed as percentages.

#### 2.3.12. Pericarp Enzyme Activities

For measuring phenylalanine ammonia lyase (PAL) activity [29], 4 g of pericarp sample was homogenized with 20 mL of borate buffer (50 mM, pH 8.5), which included 5 mM β-mercaptoethanol and polyvinylpyrrolidone (PVP) (25 g/L) and after that, the mixture was centrifuged at 15,000× *g* at a temperature of 4 °C for 20 min. The resulting supernatant served as the crude enzyme extract. To measure PAL activity, 1 mL of this extract was mixed with 1 mL of 20 mM l-phenylalanine and 2 mL of distilled water. This mixture was then incubated at 40 °C for 2 h. The reaction was halted by adding 0.1 mL of 6 M HCl, and the absorbance was recorded at 290 nm using a UV-Visible spectrophotometer (model: Mini UV 1240, Shimadzu, Kyoto, Japan). PAL activity was quantified based on the production of 1 μmol of cinnamic acid per hour, and the specific activity was reported as unit g^−1^ FW. 

To measure phospholipase D (PLD) activity [37], 5 g of pericarp sample was homogenized in 20 mL of 100 mM acetate buffer (pH 5.5), and then the mixture was centrifuged at 14,000× *g* for 10 min at 4 °C. Separately, 0.4 g of phosphatidylcholine (lecithin) was dissolved in 50 mL of ether. The solution was evaporated using a rotary evaporator at 35 °C until it was completely dry. The dried residue was reconstituted in 1 L of 100 mM acetate buffer (pH 5.5) containing 5 mM dithiothreitol and 25 mM CaCl_2_ to form the reaction substrate. For the PLD assay, 1 mL of the crude enzyme extract was mixed with 3 mL of the prepared reaction substrate. This mixture was then vigorously agitated for 1 h at 28 °C. Afterwards, the mixture was washed with petroleum ether. The aqueous phase was separated and mixed with 2 g of Reinecke salt (ammonium tetrarhodanatodiammonchromate) dissolved in 100 mL of methanol, resulting in the formation of a precipitate. The precipitate was collected by centrifugation at 16,000× *g* and redissolved in 3 mL of acetone. The absorbance of the clear supernatant was measured at 520 nm using a UV-Visible spectrophotometer (model: Mini UV 1240, Shimadzu, Kyoto, Japan), and the specific activity was reported as unit g^−1^ FW.

For measuring the activity of lipoxygenase (LOX) [38], a 5 g pericarp sample was homogenized in a chilled environment at 4 °C with 10 mL of potassium phosphate buffer (50 mM, pH 7). Then, the homogenate was centrifuged at 15,000× *g* for 15 min at 4 °C, and the supernatant was collected and analyzed for LOX activity. In this assay, 0.2 mL of supernatant was combined with 2.8 mL of same potassium phosphate extraction buffer that contained 25 mM of sodium linoleate. Then, the reaction mixture was measured at 234 nm using a UV-Visible spectrophotometer (model: Mini UV 1240, Shimadzu, Kyoto, Japan). LOX activity was quantified as the enzyme quantity required to alter the absorbance by 0.01 per minute, with specific activity reported as unit g^−1^ FW.

For measuring the polyphenol oxidase (PPO) activity [39], a 5 g pericarp sample was homogenized in 20 mL of 0.05 M phosphate buffer (pH 7.0) with 0.5 g of insoluble PVP at a cold temperature of 4 °C. The homogenate was then filtered through a muslin cloth and centrifuged at 19,000× *g* for 20 min at 4 °C. Then, the resulting supernatant was collected and used for PPO activity analysis. To measure PPO activity, 0.1 mL of supernatant was mixed with 2.9 mL of 10 mM 4-methyl catechol. The change in the reaction mixture was measured at 420 nm using a UV-Vis spectrophotometer (model: Mini UV 1240, Shimadzu, Kyoto, Japan), recorded every minute. Enzymatic activity was defined as the amount causing a 0.001 change in absorbance per minute, with specific activity reported as unit g^−1^ FW.

For measuring POD activity [40], a 2 g pericarp sample was homogenized in a mortar and pestle at 4 °C with 20 mL of 0.05 M phosphate buffer (pH 7) and 0.2 g of PVP. Then, the homogenate was strained through cheesecloth and centrifuged at 19,000× *g* for 20 min at 4 °C. The resulting supernatant was used for POD activity analysis, which involved a reaction mixture of 3 mL, comprising 25 μL of the supernatant, 2.78 mL phosphate buffer, 0.1 mL of 20 mM H_2_O_2_, and 0.1 mL of 20 mM guaiacol. The increase in absorbance was measured at 470 nm using a UV-Visible spectrophotometer (model: Mini UV 1240, Shimadzu, Kyoto, Japan) for over 2 min. One unit of enzyme activity was defined as a 0.01 absorbance change per minute, with specific activity reported as unit g^−1^ FW.

### 2.4. Statistical Analysis

All experiments were conducted in triplicate, and the results were expressed as the mean ± standard deviation. A one-way analysis of variance (ANOVA) and Duncan’s multiple range test were employed to analyze the statistical significance of the mean values. SPSS software for Windows (v12) was used for the statistical analyses in this study.

## 3. Results and Discussion

### 3.1. Pericarp Color and Appearance

**Figure 2:**
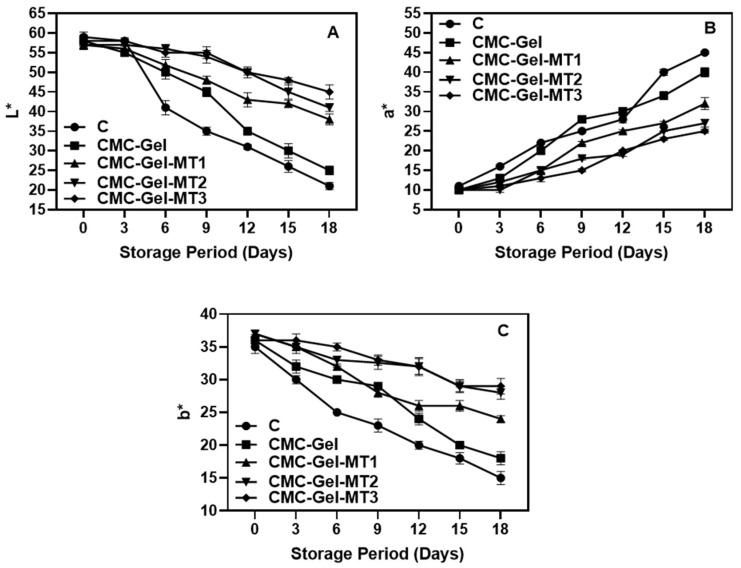
Changes in lightness (L*) (**A**), redness (a*) (**B**), and yellowness (b*) (**C**) of longkong fruit coated with or without CMC-Gel-MT at varying concentrations and stored under prolonged low temperature conditions.

**Figure 3:**
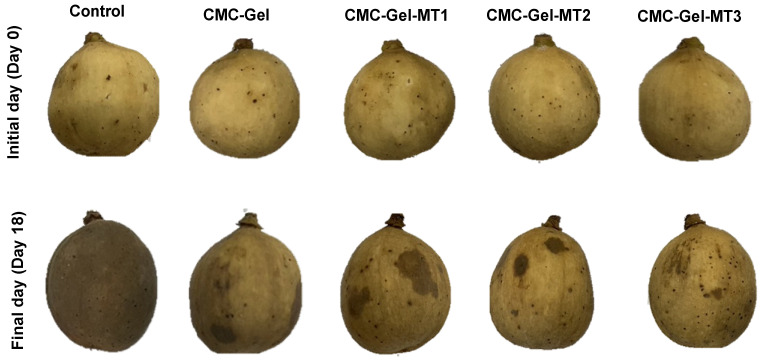
Surface observation of longkong fruit coated with or without CMC-Gel-MT at varying concentrations and stored under prolonged low temperature conditions.

Generally, color is the primary characteristic used to determine harvest maturity and purchasing decisions for longkong pericarp [7]. Changes in longkong pericarp color properties including lightness (L*), redness (a*), and yellowness (b*) of longkong fruit pericarp coated with or without CMC-Gel-MT are shown in Figure 2A–C. The overall trend of color changes in longkong fruit showed that the L* and b* values of the pericarp continuously decreased throughout storage in all samples, whereas the a* values increased in the pericarp samples during storage. Among the coated and uncoated samples, the fruits coated with CMC-Gel and CMC-Gel-MT showed better retainment of color characteristics, and furthermore, the application of MT in the coating materials provided additional protection over the control of pericarp color loss. MT performance on preventing pericarp color degradation was significantly increased when the concentration of MT increased >0.4 mM/L. Overall, significant color changes in the pericarp samples were observed after the third day of storage, continuing until storage’s end. The tropical longkong fruit and its pericarp are highly susceptible to color degradation under both warm and cold storage conditions, and particularly under cold conditions, the severity and damage are more pronounced [41]. Several studies have established that the darkening effect on the longkong pericarp under various storage conditions is mainly attributed to cellular degradation and the activation of enzymatic browning, especially by oxidoreductase enzymes including PPO, POD, and LOX [6,28]. LOX and PAL enzymes do not directly induce browning on the fruit; however, the PAL enzyme produces significant levels of polyphenolics under chilling stress, which are major substrates for PPO and POD [42]. Similarly, LOX activity increases rapidly under extended chilling conditions, using cellular materials as substrates [43]. This leads to the breakdown of the cell wall and cell membrane of the longkong pericarp, making it more accessible to PPO and POD. This aligns with findings by Lin et al. [44]. The application of CMC-based coating mitigated these changes; likely, the CMC coating acts as a barrier, preventing external environmental interactions with the fruit. Ali et al. [45] reported that application of CMC-based coating to mango exhibited significantly fewer color changes compared those untreated. Saowakon et al. [45] stated that CMC coating slightly controlled rambutan fruit browning compared to controls. Zheng et al. [46] found that the addition of melatonin boosts the fruit’s antioxidant activities, controlling lipid peroxidation and reducing cellular degradation, resulting in less pericarp browning. This study is in concordance with their findings, as the application of CMC-Gel-MT coating indeed controlled the ROS, LOX, and PPO activities in the longkong pericarp under prolonged chilling conditions. Similarly, the appearance of the CMC-Gel-MT-coated longkong pericarp on the initial day and at the end of storage days is shown in Figure 3. Photographs of the longkong pericarp were taken after the fruits were washed of the applied coating and our observation showed no visible decay. However, prolonged storage affected almost 90% of the surface with browning in the control fruit, followed by 60% of the surface showing browning in the CMC-Gel-coated fruits; on the other hand, <40% of surface browning was observed in CMC-Gel-MT-coated fruits.

### 3.2. CI Index, EL, and MDA

**Figure 4:**
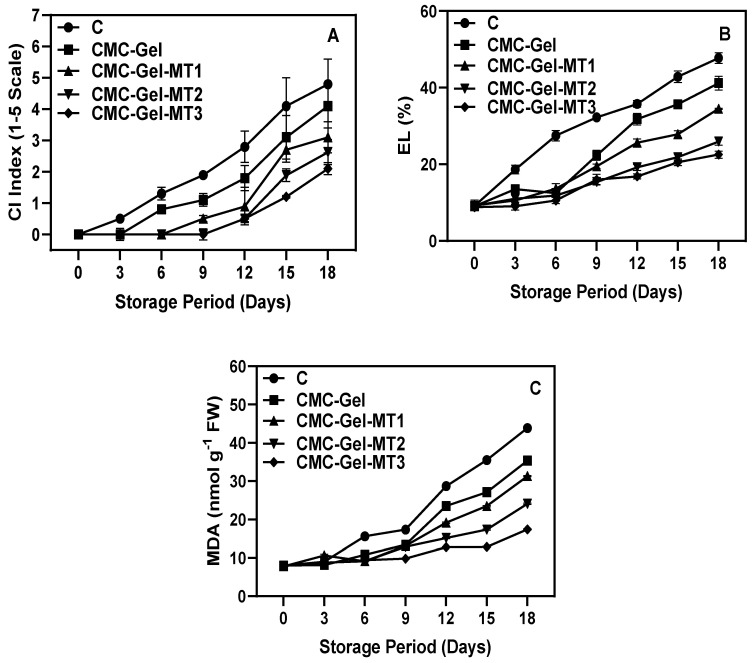
Changes in CI index (**A**), EL (**B**), and MDA (**C**) of longkong fruit coated with or without CMC-Gel-MT at varying concentrations and stored under prolonged low temperature conditions.

The CI index measures the degree of cold-induced damage in plants, assisting in the refinement of storage and handling practices to improve the quality and lifespan of produce [47]. The considerable postharvest loss of longkong fruit can primarily be attributed to the damaging effects of CI. Figure 4A displays the CI index of longkong fruit pericarp coated with and without CMC-Gel-MT. The data reveal that the onset of the CI in the examined fruit pericarp varied significantly across the different sample types. CI began to appear in control samples at the beginning of the third day of storage, while CMC-Gel-MT-treated samples showed slightly visible symptoms after the ninth day of storage, and they tended to further intensify with extended storage days. The control of CI incidence using CMC-Gel-MT was MT dose-dependent, with higher concentrations providing better control against CI incidence compared to lower concentrations and control. The CI in control samples increased intensively as the storage period increased. Generally, the longkong fruit pericarp is highly susceptible to CI. Its trichomes on the pericarp surface can act as a signaling agent, inducing an adverse response (such as the production of oxidoreductase enzymes and reactive oxygen species) against stressful environmental conditions, particularly chilling temperatures. The elevated production of reactive oxygen species results in oxidative damage to cellular membranes, ultimately compromising membrane stability [48]. This deterioration often presents as an increased level of electrolyte leakage compared to normal conditions (see Figure 4B). The utilization of edible coatings, especially CMC-Gel and CMC-Gel-MT, can enhance preservation by stabilizing the membrane structure and stability of horticultural produce. This, in turn, reduces electrolyte leakage and extends the shelf life of coated fruits [19,49].

The changes in EL level in the pericarp of longkong fruit coated with Gel-CMC-MT at different concentrations are shown in Figure 4B. The results suggest that the extended storage at low temperatures significantly increased the EL level in all sample types. Furthermore, the severity of the leakage seems to augment with the duration of storage. Comparatively, longkong pericarps coated with CMC-Gel and CMC-Gel-MT exhibited a substantial reduction in leakage compared to the noncoated control samples. In comparison with CMC-Gel and its MT variation, the CMC-Gel-MT showed better control of EL and, furthermore, MT2 and MT3 samples exhibited better EL control in the fruits as compared to their low-concentration variation. EL is regarded as a key identifier of chilling damage in fruits [50]. Electrolytes in living cells reside within compartments bound by membranes, and when these cells encounter stress factors like chilling temperatures that can cause cold damage, or during natural aging, the proteins and lipids within the membranes undergo degradation and oxidation [51]. This leads to modifications in their structure, which in turn weakens their integrity and increases membrane permeability. The present study demonstrates that an escalation in EL is an indicator of increased cold injury in longkong pericarp. Low-temperature-induced membrane damage was noticeably significant after just 6 days of storage, possibly due to biochemical changes occurring within the bilayers following the initial chilling temperature storage. However, the addition of MT in the CMC-Gel coating significantly controlled those changes and reduced the chilling injury impact on the fruit’s pericarp. 

MDA is an important indicator of membrane dysfunction, and its increasing level corresponds to an increase in oxidative damage and membrane degradation [52]. The MDA content in the CMC-Gel-MT-coated and uncoated longkong pericarp continuously increased in trend (Figure 4C). Control fruits had the predominant level of MDA accumulation in their pericarp, followed by the treated samples. However, among the treated ones, the higher concentration of CMC-Gel-MT in the treated samples had the lowest MDA level in their pericarp (*p* > 0.05). However, when compared with other samples in this study, MT2 and MT3 were highly significant in controlling the MDA content (*p* < 0.05). CMC-Gel-MT coating may be able to suppress the membrane damage by altering the fatty acid arrangements and limiting the ROS production in the cell membrane. Composite coating made of protein and polysaccharide in the presence of crosslinkers creates strong interactions via covalent bonding, which helps reduce structural degradation, water loss, and maintain the firmness of fruits [53,54]. Saowakon et al. [45] found that the CMC-based composite coating increased the vitamin C content in rambutan fruit, which is one of the strong antioxidant vitamins that can alleviate the severity of lipid oxidation plants.

### 3.3. Fruit Weight Loss and Respiration Rate

**Figure 5:**
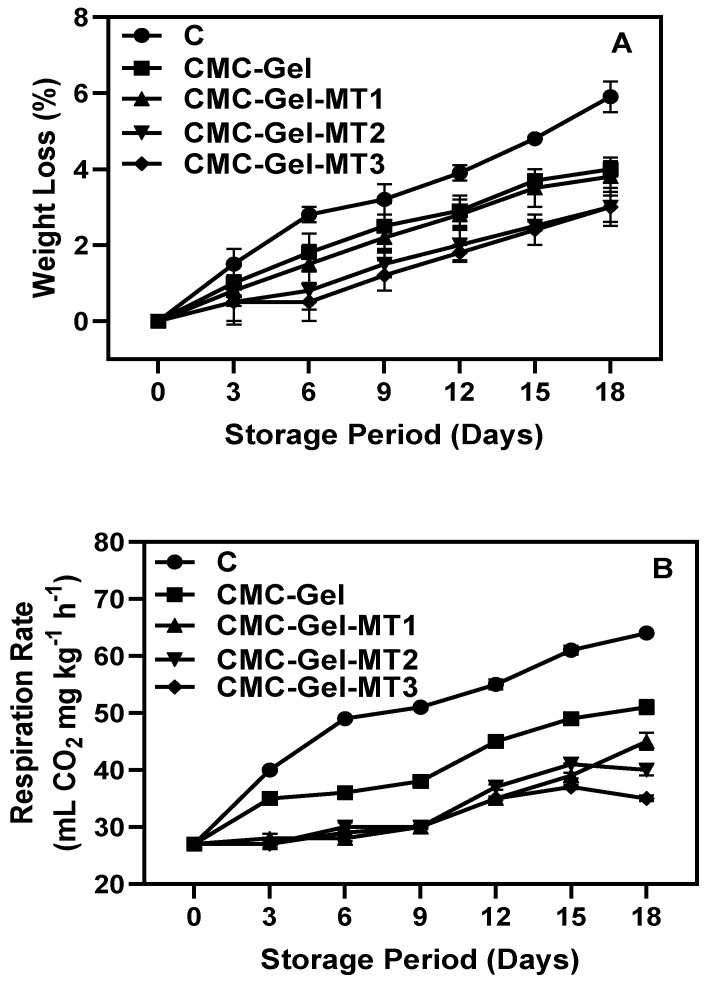
Changes in weight loss (**A**) and respiration rate (**B**) of longkong fruit coated with or without CMC-Gel-MT at varying concentrations and stored under prolonged low temperature conditions.

The weight loss of the longkong fruit pericarp increased steadily over the storage period in all samples, as depicted in Figure 5A. Notably, the control fruits, which were not coated, exhibited particularly severe weight loss. On the other hand, the longkong fruits coated with CMC-Gel-MT displayed significantly less weight loss. The extended low-temperature storage period induced physiologically based weight loss via endogenous biochemical activities that occurred due to CI severity, whereas the CMC-Gel and CMC-Gel-MT coatings had a significant impact on the adverse changes and controlled the sample’s weight loss. The results show that a sharp increase in weight loss of longkong fruit, particularly in the control group, was found on the third day and the trend continued until the end of the storage. While the CMC-Gel-MT-coated samples did exhibit some weight loss, it was significantly less than that observed in the control group. Among the different CMC-Gel-MT-coated samples, the differences in weight loss were not significantly higher, especially in the MT2 and MT3 samples. Samples that were coated with CMC-Gel and CMC-Gel with low MT concentrations showed slightly lower performance in controlling the weight loss. Generally, the loss of fresh mass in longkong fruit pericarp could lead to visible symptoms, particularly dryness and browning on the pericarp surface, which could result in significant economic loss [6,55]. In fresh produce, moisture loss is critical to maintaining the weight of the product [56]. Stress-induced respiration might induce a higher transpiration rate in the fruits, which also might contribute to weight loss [57]. Continuous moisture loss in food may result in the desiccation of the product, rendering it unsuitable. Coating creates barriers in fresh produce and could control the impediment of water vapor transmission by covering the surface and any minor cracks, ultimately reducing moisture loss [19,58]. Although the CMC-Gel-MT-based coating could control weight loss, a slight increase in weight loss was still observed during storage. This could be due to the formation of a semipermeable layer between the coated fruit and storage conditions.

The respiration rate is a dependable measure for tracking the quality of fruit during storage, and alterations in this rate can substantially reflect in the fruit’s quality and shelf life [59]. The respiration rate of the longkong fruit coated with or without CMC-Gel-MT were tested, and the results are shown in Figure 5B. The present study found that the fruit’s overall respiration rate tended to increase in the samples all over the storage. The respiration rate in the CMC-Gel-coated samples was controlled slightly better than in the control. Furthermore, incorporating a higher concentration of MT into the coating showed better overall performance in controlling the respiration rate. Generally, changes in the respiration rate of fruit reflect the changes in the physiological state of the fruit and indicate the advancement of the senescence process [60]. Normally, the chilling injury in tropical fruits leads to a sharp increase in respiration rate, and this increase is often a stress response from the fruit to chilling conditions; however, when the chilling stress is prolonged for a long period, the cellular functions could be impaired in the fruit and eventually decrease the respiration rate [61]. This is in accordance with the present study. Our study results showed that longkong pericarp treated with CMC-Gel-MT significantly controlled the rate of respiration, exhibited minimal changes in respiration rate, and maintained its physiological state better than in the control. Ozden and Bayindirli [62] reported that edible coatings on fruits could lead to accumulation of high CO_2_ and low O_2_ concentrations in the interstitial space between the coating and the fruit surface, potentially decreasing the respiration rate.

### 3.4. Production of Reactive Oxygen Species

**Figure 6:**
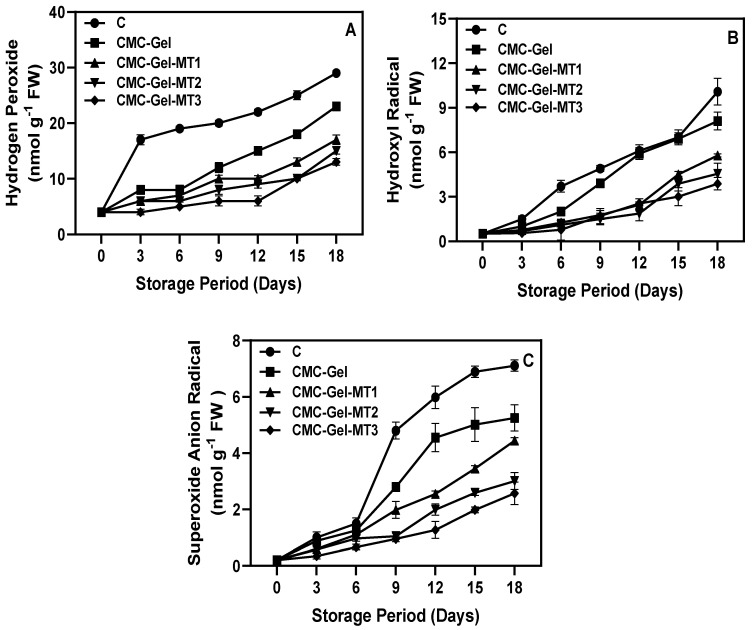
Changes in hydrogen peroxide (**A**), hydroxyl radical (**B**), and superoxide anion radical (**C**) of longkong fruit coated with or without CMC-Gel-MT at varying concentrations and stored under prolonged low temperature conditions.

Changes in reactive oxygen species (ROS), including hydrogen peroxide (H_2_O_2_), hydroxyl radical (OH^−^), and superoxide radical anion (O_2_^−^), in CMC-Gel-MT-coated and uncoated longkong fruit stored under prolonged chilling stress are depicted in Figure 6A–C. Generally, CI induces strong oxidative stress in horticultural produce, leading to increased ROS production, which in turn causes irreversible damage to macromolecules [61]. H_2_O_2_ is a potent free radical that inflicts oxidative damage on fresh produce, thereby accelerating aging and reducing shelf life [63]. H_2_O_2_ levels in the pericarp of CMC-Gel-MT-coated and uncoated longkong fruits were observed to continuously increase under prolonged chilling stress. However, these levels were significantly lower compared to those in fruits with only the CMC-Gel coating and the control group. Among the MT samples, those with a higher concentration were more effective in scavenging H_2_O_2_. The overproduction of H_2_O_2_ under low-temperature conditions can be detrimental due to the resultant excessive free radicals that damage cell membranes, typically caused by an imbalance between free radical production and antioxidant levels. Similarly, the levels of OH^−^ and O_2_^−^ radicals also tended to rise in longkong fruit during extended storage under chilling conditions, with O_2_^−^ radical production surpassing that of the other two ROS. CMC-Gel-MT at various concentrations succeeded in minimizing the accumulation of OH^−^ and O_2_^−^ radicals. This finding aligns with the study by Mirshekari et al. [64], who discovered that MT treatment on sapota fruit effectively regulated ROS production compared to controlling fruits. The application of CMC-Gel-based coating to the fruit surface strengthens the membrane by deeply penetrating the pores and cracks, thereby preserving cell membrane integrity and consequently reducing H_2_O_2_ production. Besides its ROS scavenging capacity, MT also bolsters the activity of the ROS scavenging system, not only by stimulating the expression of ROS scavenging genes and enhancing the enzyme activity but also by reducing LOX activity and generating additional ascorbic acid [65]. These actions contribute to maintaining membrane integrity, as evidenced by the decreased accumulation of MDA [66]. In addition, MT can regulate ROS signaling pathways, such as the MAPK and Nrf2 pathways, and these pathways play a role in regulating the production and detoxification of ROS [67,68]. MT has also been shown to induce the expression of heat shock proteins (HSPs) in fruits. HSPs are a group of proteins that are expressed in response to stress, and they can help to protect cells from damage caused by ROS and other stressors [69]. Furthermore, the alleviation of ROS in longkong pericarp can also be achieved by their secondary metabolic product, particularly phenolic compounds, which is rich in longkong [28,70]. Phenolic compounds exhibit antioxidant properties by directly scavenging the ROS by themselves and/or chelating transition metals, predominantly iron and copper, through their hydroxyl and carboxyl functional groups, thereby mitigating oxidative stress [71]. Recent studies have reported that phenolic acids are able to upregulate the expression of antioxidant enzymes, such as superoxide dismutase, catalase, and peroxidase activities, thus controlling ROS production and accumulation in the chilling-injured plant crops [72].

### 3.5. Pericarp PAL Enzyme Activity, Phenolic Content, and Antioxidant Activities 

**Figure 7:**
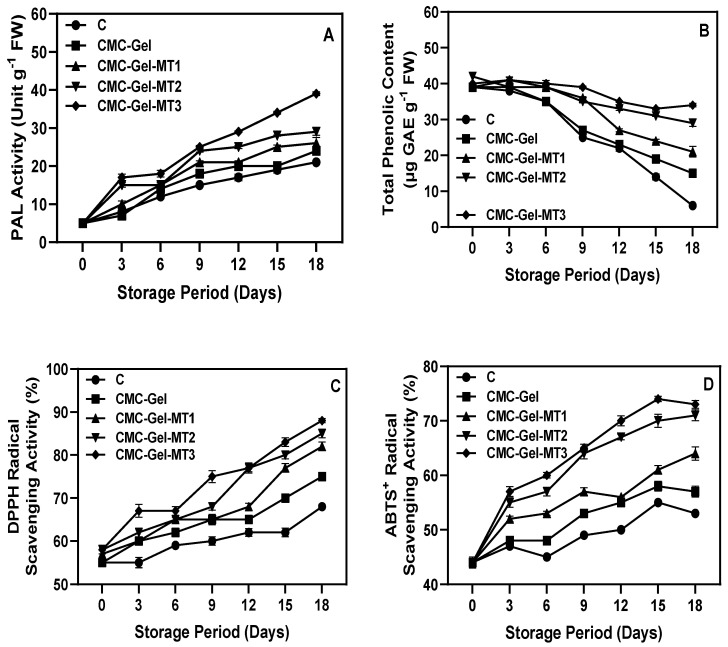
Changes in PAL activity (**A**), total phenolic content (**B**), DPPH radical scavenging activity (**C**), and ABTS^+^ radical scavenging activity (**D**) of longkong fruit coated with or without CMC-Gel-MT at varying concentrations and stored under prolonged low temperature conditions.

The biosynthetic phenylpropanoid pathway can be readily triggered in plants under abiotic stress, especially under extreme temperature conditions, both high and low, and this activation leads to the buildup of phenolic compounds. PAL is the pioneer enzyme in the phenolic metabolism of plants, which generates phenolics in the plants via the phenylpropanoid pathway [73]. PAL catalyzes the conversion of phenylalanine to trans-cinnamic acid as well as other varieties of phenolic compounds including flavonoids, coumarins, hydrolysable tannins, monolignols, lignans, and lignin [74]. This study showed that CMC-Gel-MT-coated and uncoated longkong fruit pericarp samples showed an increase in PAL activity with fluctuations (Figure 7A). In comparison with control and CMC-Gel-coated samples, MT-incorporated (MT1–4) samples showed increased PAL activity. The higher MT concentration in the coating emulsion significantly increased longkong pericarp PAL activity. This is in accordance with the study by Esmaeili et al. [75], who reported that an increase in MT concentration positively increased the PAL enzyme and, consequently, abundance of phenolic acids produced. Studies have reported that increased PAL activity by MT is mainly due to enhanced antioxidant activity as well as an upregulation of the genes that encode PAL [76]. Similarly, the phenolic contents in the plants tend to increase as the abiotic stress level increases, which is an indicator for stimulus response to scavenge or prevent stress-induced damage in plants, particularly by reactive oxygen species [77]. The present study showed that the phenolic content in longkong pericarp tended to decrease gradually throughout storage, regardless of sample type. However, CMC-Gel-MT-coated pericarp samples had significantly higher phenolics, in accordance with the PAL activity in the samples (Figure 7B). On the other hand, the phenolic contents of the CMC-Gel and control samples were lower than in the CMC-Gel-MT samples, but the CMC-Gel samples were slightly better than the control. The changes in phenolic level in pericarp could be attributed to chilling stress, oxidation, and antioxidant properties. The phenolic content in the MT-coated samples was directly in concordance with the amounts of MT concentrations. Plant phenolics play a crucial role in controlling abiotic stress, as this stress could disturb the balance between ROS generation and the acceleration of ROS-induced damage in vital macromolecules such as nucleic acids, proteins, carbohydrates, and lipids [78]. Plant phenolics act as physiological chemical messengers that inhibit the catabolic reactions in plants through stress activation of various symbiotic processes [79]. In general, longkong pericarp is very sensitive due to surface trichomes, and always tends to produce more phenolics than usual due to accidental stimuli, which is sometimes a drawback, as it might unwantedly induce browning; however, other than that, it possesses excellent antioxidant properties. Longkong pericarp polyphenolics may be able to scavenge stubborn free radicals, especially when produced under extended chilling stress [28]. The antioxidant activities, mainly DPPH radical and ABTS radical scavenging activities of CMC-Gel-MT-coated and uncoated longkong results, are shown in Figure 7C,D. Both radicals were significantly controlled in longkong pericarp using the tested coating; however, in comparison with ABTS radials, the coating material exhibited better scavenging efficiency against DPPH radicals. The antioxidant potential of uncoated longkong fruit pericarps initially increases but gradually decreases during storage, leading to browning. This decrease in antioxidants corresponds to elevated ROS levels (O_2_ and H_2_O_2_), causing membrane damage, as evidenced by increased MDA content and charge leakage [80]. Enzymes PPO and POD catalyze the oxidation of phenolic compounds, further promoting browning. The reduced antioxidant efficiency accelerates ROS accumulation, intensifying membrane oxidative damage and resulting in brown longkong fruit pericarps during storage [81]. Furthermore, the increased MT concentration in the coating emulsion significantly increased the scavenging efficiency of the longkong fruit against radicals. The antioxidant potency in longkong pericarp is mainly attributed to its polyphenolic contents [82]. This study proves that the application of MT in coating emulsion increases PAL activities and consequently increases secondary metabolic products, particularly phenolic content and antioxidant potency.

### 3.6. Lipid Metabolism-Based Enzyme Activities 

**Figure 8:**
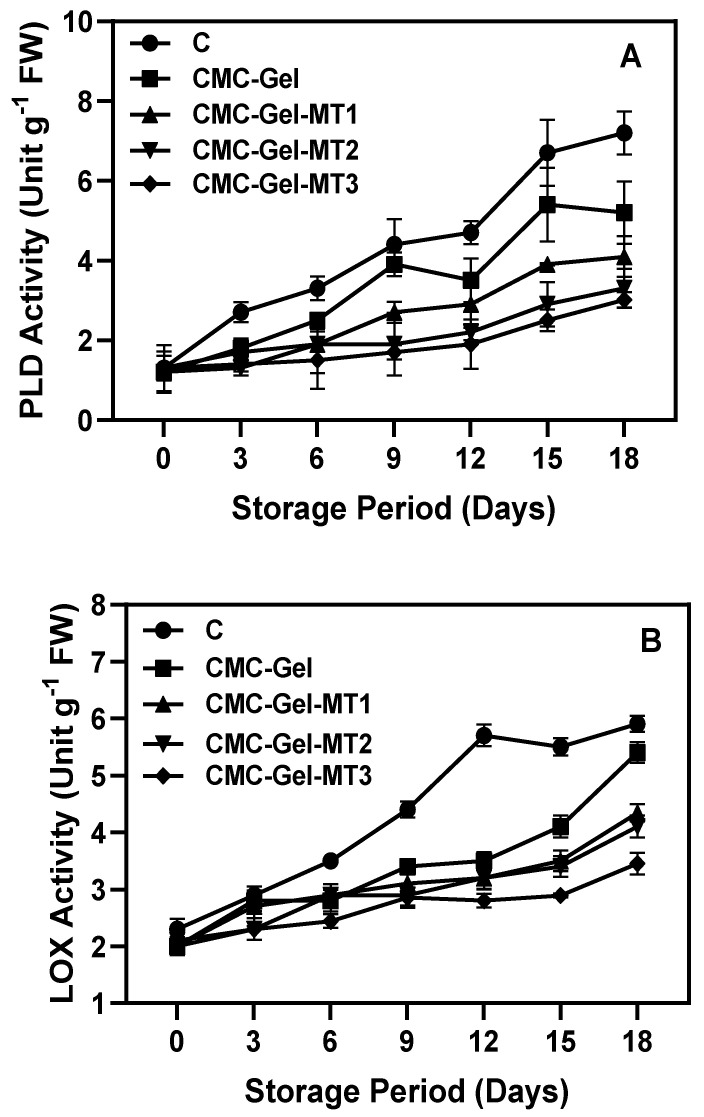
Changes in PLD activity (**A**) and LOX activity (**B**) in longkong fruit coated with or without CMC-Gel-MT at varying concentrations and stored under prolonged low temperature conditions.

PLD and LOX are crucial enzymes involved in plant senescence and stress-induced deterioration in fruits; furthermore, their activities notably intensify in fruits and vegetables subjected to prolonged chilling stress [83]. By catalyzing the oxidation of unsaturated fatty acids, both enzymes contribute to membrane lipid peroxidation, significantly impacting maturation, senescence, and stress responses in organisms [84]. Specifically, LOX facilitates the formation of oxylipins from polyunsaturated fatty acids, such as linoleic and linolenic acid, which are vital for plant development and resilience against various stresses [85]. This study tested the LOX and PLD activities in coated and uncoated longkong fruit pericarp during storage under extended chilling stress (Figure 8A,B). The results showed that despite the samples tested, LOX and PLD levels continuously increased in the fruit pericarp throughout storage. The severity of the increment in the tested activities were noticed after the fourth day of storage in control samples for PLD activity and sixth day of storage for LOX activities. For the CMC-Gel and CMC-Gel-MT samples, the onset of mild severeness in the enzyme activities was noticed after 8 days of storage. Among the samples, the CMC-Gel-MT-coated longkong pericarp was able to control the intensity of enzyme activities, followed by the CMC-Gel and control samples. The control samples showed elevated activity of these enzymes, indicating extreme cell membrane phospholipid degradation. On the other hand, the CMC-Gel-MT-coated samples efficiently controlled lipid metabolism-based enzyme activities, the efficiency of MT in controlling such enzyme activities was dose-dependent, and higher MT concentration effectively controlled those enzyme activities. CMC-Gel samples performed slightly better than the control samples, which might be due to the ability of structural strengthening by the coating polymers. Magri et al. [86] tested the efficiency of CMC-based composite coating, and their study effectively controlled the LOX activity in the minimally processed apple fruit under chilling conditions. MT ability to interact directly with the hydrophobic lipid phase of cell membranes due to its lipid solubility and potential to alter PLD functionality may be key factors in controlling PLD activity and pericarp cellular damage. MT contributes to the reduction in LOX activity mainly through its antioxidant properties, modulation of gene expression, direct regulation of enzyme activities such as PLD, and general alleviation of stress responses in plants [87]. However, MT has a positive effect on controlling PLD and LOX activities, but the exact mechanism can be complex and may involve a combination of various factors, possibly varying among different plant species or under different types of stress. 

### 3.7. Browning Related Enzyme Activities

**Figure 9:**
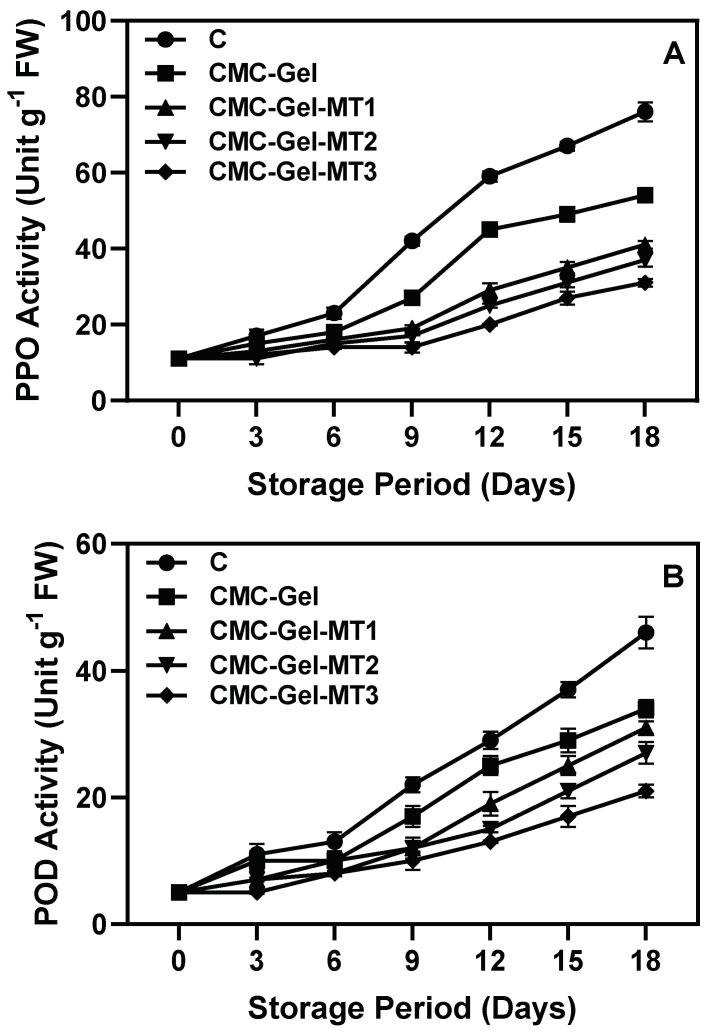
Changes in PPO activity (**A**) and POD activity (**B**) in longkong fruit coated with or without CMC-Gel-MT at varying concentrations and stored under prolonged low temperature conditions.

PPO and POD are the primary enzymes responsible for oxidizing phenolic content in the pericarp of longkong fruit, leading to browning during extended storage and prolonged exposure to stress [88]. The current study demonstrated that extended storage under continuous chilling stress significantly increased PPO and POD activities in the pericarp of longkong fruit, regardless of sampling variability (Figure 9A,B). However, the severity of PPO and POD activities was markedly reduced in fruit pericarps coated with CMC-Gel-MT at various concentrations, followed by those coated with CMC-Gel. The uncoated control samples exhibited the highest PPO and POD activities. Although both PPO and POD activities continually affected the longkong fruit pericarp, PPO activity was more predominant compared to POD. Studies have reported that an increase in PPO induces more browning in longkong pericarp compared to POD activities [41]. Increases in POD enzyme activity is also associated with the accumulation of H_2_O_2_ in the fruit pericarp, as POD utilizes H_2_O_2_ as a co-substrate alongside its organic substrate, phenolic acid [89]. Generally, the POD enzyme acts as a secondary stimulator of the browning reaction by converting the phenolic substrate into quinones, since the activation of POD requires two substrates [90]. In contrast, PPO rapidly reacts with phenols in the presence of oxygen. Both PPO and POD produce quinones, which are further polymerized into melanin, the browning agent that adversely affects the fruit’s marketability and shelf life [91]. Xiao et al. [92] reported that MT treatment significantly downregulated the expression of PPO (PPO1, PPO2, PPO3, and PPO4) and POD (POD1, POD4, POD72, and POD-N) genes in fresh-cut taro. Wei et al. [93] found that MT treatment helps protect against subcellular decompartmentalization in rambutan pericarp, thereby reducing enzymatic browning. Magri et al. [86] reported that LOX-driven lipid peroxidation produces MDA and H_2_O_2_, leading to cellular degradation and fruit discoloration. However, highbush blueberries treated with melatonin exhibit reduced MDA and H_2_O_2_, suggesting minimized cellular harm and browning from decreased PPO and LOX activities during cold storage. This aligns with the present study, which noted reduced MDA and H_2_O_2_ levels in longkong samples coated with CMC-Gel-MT.

## 4. Conclusions

The application of CMC-Gel and CMC-Gel-MT coatings on longkong fruit stored under extended chilling conditions had significantly enhanced postharvest preservation through a series of adaptable biochemical and physiological mechanisms. These coatings effectively inhibited the activity of key enzymes responsible for browning, namely, polyphenol oxidase and peroxidase. This enzymatic suppression played a pivotal role in mitigating color deterioration and chilling injury symptoms. Additionally, the formation of a semipermeable barrier by these coatings was instrumental in reducing moisture loss from the fruit. CMC-Gel-MT coating regulated the respiration rate of longkong fruit, effectively decelerated the senescence process, and prolonged shelf life. Furthermore, the CMC-Gel-MT coating effectively controlled the extensive accumulation of reactive oxygen species in longkong pericarp. The CMC-Gel and CMC-Gel-MT coatings contributed to maintaining the structural and functional stability of the fruit by modulating the activities of phospholipase D and lipoxygenase. Overall, this study showed that addition of melatonin in the coating significantly improved the longkong fruit’s quality; furthermore, the action of MT on the longkong fruit was dose-dependent, and higher concentrations performed better than the lower concentrations. Concentrations of 0.8 and 1.2 mM/L MT showed similar effects on the longkong fruits and therefore this study recommends the use of a 0.8 mM/L concentration for optimal performance regarding shelf life enhancement and quality retention of longkong fruit. In summary, CMC-Gel-MT coatings present a promising approach in the realm of postharvest fruit treatment technologies, offering practical and effective means to address the challenges of fruit preservation during storage. The application of CMC-Gel-MT coating to fresh longkong fruit shows promise for commercial production processes. This method effectively maintains quality and extends the shelf life of longkong fruits. Additionally, it is a cost-effective and environmentally friendly treatment.

## Data Availability

Data is contained within the article.
